# An evaluation of the performance of medical helmets used in healthcare for the protection of vulnerable patients

**DOI:** 10.3389/fbioe.2025.1575075

**Published:** 2025-04-16

**Authors:** Rory England, Marina Haynes, Harry Mee, Jon Farmer

**Affiliations:** ^1^ Sports Technology Institute, Woldson School of Mechanical, Electrical and Manufacturing Engineering, Loughborough University, Loughborough, United Kingdom; ^2^ Division of Rehabilitation Medicine, Department of Clinical Neurosciences, University of Cambridge and Cambridge University Hospital, Cambridge, United Kingdom

**Keywords:** brain injury, brain surgery, helmet, head impact, rehabilitation, craniectomy, fall injury

## Abstract

**Introduction:**

Medical helmets (MHs) are used by individuals with an increased vulnerability to falls and are essentially unregulated in the UK; therefore, their impact performance is unproven. This study investigated the performance of a selection of medical helmets available to clinicians using general techniques to determine their protective performance against impacts. Additionally, clinicians have stated that medical helmets need to consider focal vulnerabilities to impact (often a postsurgical site of a decompressive craniectomy); therefore, novel techniques were specifically employed for measuring the protection of a focal site.

**Materials and Methods:**

A freefall drop test methodology was used to assess six medical helmets (MH1–6) and two sports helmets (SH1 and SH2). The headform was instrumented with six degrees of freedom instrumentation to quantify global kinematics metrics related to injury risk (peak linear acceleration (PLA), peak angular velocity (PAV), peak angular acceleration (PAA), head injury criterion (HIC), and brain injury criterion (BrIC)), and a thin-film contact pressure measurement system was used to quantify the contact area (above a threshold of 560 kPa) focal to the impact. Due to the advanced nature of these measurements, a novel biofidelic headform was used to more accurately represent local deformation. Additionally, impact performance was plotted against two proxy measures of comfort.

**Results:**

The difference in performance between the worst and best helmets ranged from 90% to 2844%, showing a substantial variation. HIC, PLA, and PAA showed the largest range, whereas PAV showed the smallest range. Nonetheless, there was good agreement between each kinematic metric regarding the rank order of the medical helmets. The contact pressure was a consistent outlier. Each metric included at least one injury threshold, which MH4 and MH6 consistently exceeded (15/18 occasions).

**Discussion:**

MH2 and MH3 were the only medical helmets comparable to sports helmets in terms of both comfort and performance. MH1 showed excellent performance metrics but exhibited possible discomfort, while MH4 was above average across both measurement categories. MH4 and MH6 were significantly deficient compared to the sample of helmets. These results highlight the need for standardisation.

## Introduction

In healthcare, medical helmets (MHs) can be prescribed by clinicians for the protection of patients who are vulnerable to falls (Mee et al., 2022a). This includes individuals with medical conditions that increase their likelihood of falling and individuals with medical conditions that may increase the outcome severity of a fall such as those with poorly controlled epilepsy or patients with skull defects, including those who have undergone a decompressive craniectomy (DC). In the UK, the total estimated economic burden attributed to falls is £4.4 billion (Department of Health and Social Care, 2019). The impact of a head injury on a patient’s function and quality of life can be life-changing, with significant economic consequences; therefore, the use of effective MHs has the potential to reduce this impact and the economic burden associated with these falls.

DC is a surgical procedure in which a portion of the skull bone is removed to relieve pressure from brain swelling in the intracranial vault (Mee et al., 2022b), most commonly following a traumatic brain injury or stroke. DC is reported in up to 13% of emergency neurosurgical procedures (Silva et al., 2020) and is often used as a last resort to prevent death (Mee et al., 2022b). Patients who undergo DC experience an increased risk of falling and an increased potential for severe injury from falls. Due to this increased risk, it is common for clinicians to prescribe MHs for DC patients post-operation (Mee et al., 2022a). However, MHs are not subjected to a standardised impact attenuation test, and clinicians lack quantitative evidence to make informed decisions regarding the optimal MH for each scenario. Scientific literature related to this topic is limited to two studies (Martel et al., 2021; Barrett et al., 2022), which have applied rudimentary helmet test standard methodologies to benchmark MH performance. Neither of these studies has investigated efficacy with respect to specific mechanisms of increased risk from falls, such as the risks faced by DC patients nor have they evaluated products available to healthcare providers in the UK. Therefore, the aim of this research was to assess the efficacy of MHs available to UK clinicians, with specific consideration given to post-DC surgery as a common but unique use case.

## Materials and methods

### Impact testing method

**FIGURE 1:**
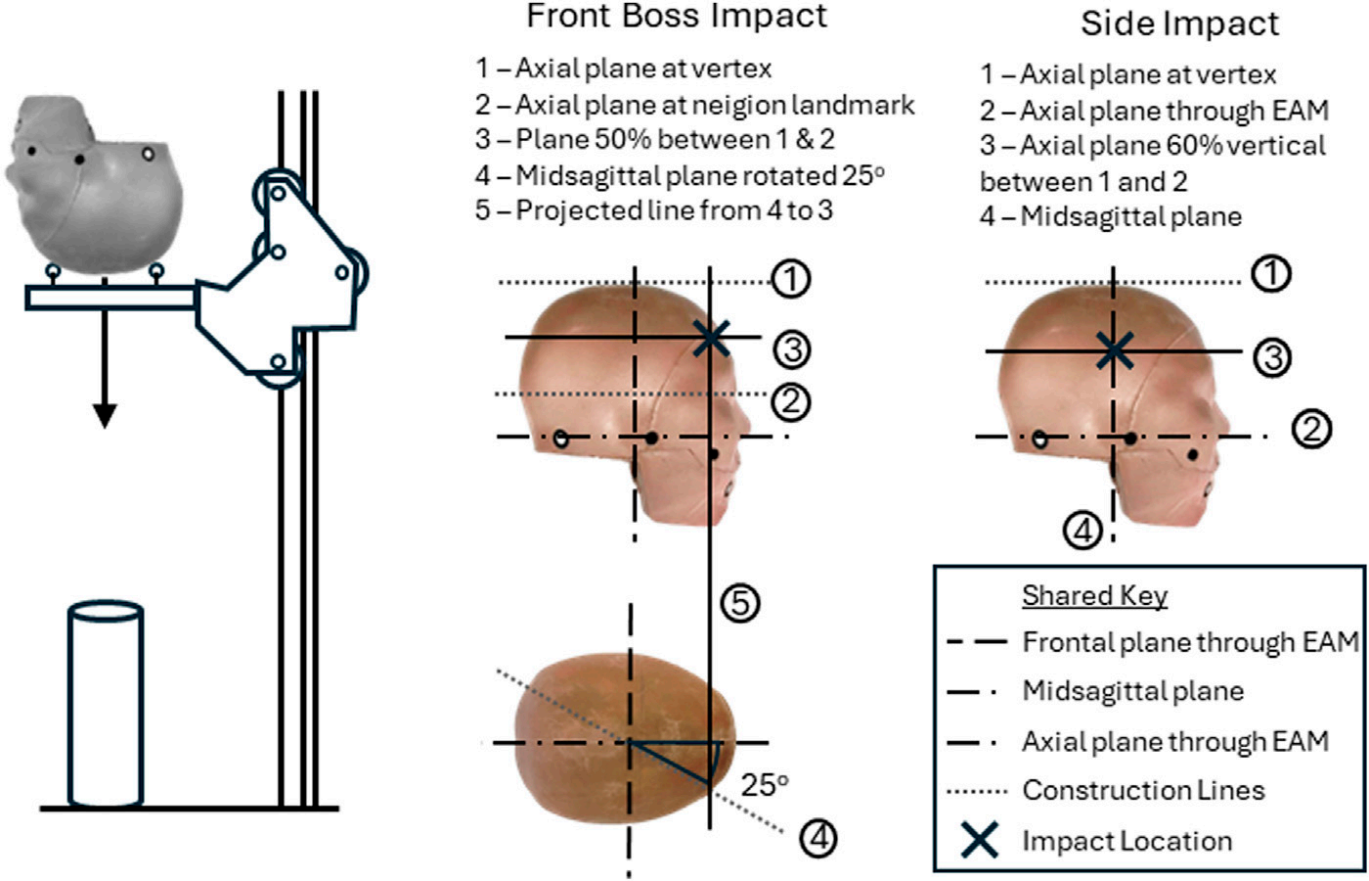
Diagrams of the drop test methodology and anatomical definition of impact locations.

Testing was conducted using the freefall drop test methodology (Figure 1). A helmeted and instrumented headform was placed on a cradle, raised to a specified height, and dropped under gravitational free fall onto a flat anvil. On contact, the cradle passed over the anvil, and the head was free of constraints. Laser gates were used to measure the preimpact velocity. One drop height was used (0.6 m), specified as the common height of a fall from a hospital bed (Morse et al., 2015), which is in agreement with a previous study (Martel et al., 2021). Two test locations were defined relative to anatomical landmarks: the front boss and the side, as shown in Figure 1. The impact vectors were approximately normal at these points. These were two common DC surgery locations (Moon and Hyun, 2017). Each impact was repeated three times, including at least one impact with an entirely new helmet, and never on a previously used location.

### Headform and instrumentation

A custom-built headform developed at Loughborough University, namely, LU 1.1 (England, 2025), was chosen due to its high biofidelity compared to commonly used headforms, such as Hybrid III. LU 1.1 is much more representative of the 50th percentile male in terms of material stiffness properties, centre of mass locations, moments of inertia, overall mass, and geometric properties. This headform was chosen because its properties allow it to exhibit a more realistic response and local contact pressure. The headform was instrumented with a six-degrees-of-freedom accelerometer and gyroscope (SLICE NANO hardware with a 6DX Pro sensor, DTS) (California, United States) to capture the impact kinematics (angular velocity and linear acceleration) of the head. This was sampled at 20 kHz.

Considering the specificity of the DC injury mechanism, the surface of the head was instrumented with an F-Scan thin-film pressure sensor (Tekscan, MA, United States). The objective of this study was to measure the local distribution of the force applied directly to the DC surgery location, which is not directly captured in global head kinematics. The range of this sensor was up to 575 kPa, and the sampling rate was 750 Hz. The pressure sensor was applied to a total area of 143.2 cm^2^, which spanned across the frontal and parietal bones along the superior border of the temporal and sphenoid bones to cover two 6.5 × 6.5 cm potential DC sites. The decision to instrument the surface of the headform highlighted the importance of using a biofidelic headform.

### Data processing and analysis

Linear acceleration and angular velocity time histories from DTS 6DX Pro were processed using MATLAB (Natick, MA, United States). A Butterworth low-pass filter of 1,650 Hz was applied to the linear acceleration and angular velocity data, and a 300-Hz filter was separately applied to the angular velocity data before it was differentiated to provide angular acceleration in line with standard practice (Martin et al., 1998). Peak linear acceleration (PLA), peak angular velocity (PAV), peak angular acceleration (PAA), head injury criterion (HIC), and brain injury criterion (BrIC) were calculated to measure the overall risk of head injury based on global kinematics. Three metrics were extracted directly from the kinematic traces (PLA, PAV, and PAA), and three were calculated using established equations in the literature [BrIC (Takhounts et al., 2013), HIC (Newman, 1980), and DAMAGE (Gabler et al., 2019)].

The contact pressure data were analysed using a combination of native software and MATLAB. Pressure magnitude was presented in three classes based on pain pressure threshold (PPT) values. PPT values for most regions of the body range from 210 to 510 kPa, depending on the individual and anatomical location (Maquet et al., 2004; Jones et al., 2007; Trueba and Gasparini, 2021; Evans et al., 2024). For individual subjects, bruising has been observed for PPTs in the range of 600–1,200 kPa (Jones et al., 2007). Within this context and the delicate nature of brain tissue, low impact pressure was defined as <160 kPa, moderate impact pressure was defined as 160–560 kPa, and high impact pressure was defined as >560 kPa. Contact pressure data included a 22 kPa noise threshold and a nearest-neighbour averaging algorithm to reduce noise. In each presented figure, helmet performances were grouped using a k-means clustering algorithm (David and Vassilvitskii, 2007) into weakest-performing (red), mid-performing (yellow), and best-performing (green) helmets, represented with colour coding.

### Helmet selection and characterisation methods

Six exemplar MHs were selected for this study. These helmets are typical of those available to clinicians in the eastern region of England. For commercial reasons, these are referred to as MH1–MH6. Two low-cost sports helmets were selected for comparison, namely, a soccer head protection product (SH1) and a cycling helmet (SH2). Each helmet was fitted to the headform based on the manufacturer’s recommendations to ensure correct coupling between the helmet and the head. Each helmet was characterised to enable impartial discussion based on construction rather than the manufacturer and model. The mass of each helmet was recorded using Mattler Toledo SB8001 scales with an uncertainty of ±0.5 g (Columbus, United States). The construction type was broken down into shell material and energy-absorbing material (foam). The shell and foam were each given a verbal descriptor, and shore O hardness measurements were taken for soft foams (e.g., PU or EVA) but omitted for hard foams (e.g., EPS). For helmets that omitted a distinct shell material, Shore hardness was calculated as the average of three internal and three external measurements. Thickness measurements were taken for the foam at the front and side with micrometres (LINEAR, Middlesex, UK) and calculated as the average of five repeats in each region. A measure of discomfort in the fit of each helmet was quantified as the contact area between the helmet and head above a threshold of 60 kPa after fitting the helmet (measured across the instrumented region). This was defined as the ‘discomfort area’ and was reported in cm^2^. This threshold represents the conservative threshold for the onset of ‘discomfort,’ which is used in NFL impact testing to determine which headform correctly fits a given helmet (Jadischke et al., 2013).

### Net performance and net comfort ratings

All metrics were combined into a net performance rating and a net comfort rating per helmet on a scale of 0–100. On this scale, 0 represents two standard deviations worse than the mean of this study and 100 represents two standards better than the mean. The net performance rating considered all performance metrics (PLA, PAV, PAA, HIC, BrIC, DAMAGE, and impact contact pressure), and the comfort rating considered mass and discomfort area after fitting.

## Results

### Pre-testing helmet characterisation

Table 1 presents characterisation results for the eight helmets tested. This includes descriptors of helmet construction type and measurements of mass, material Shore hardness, foam thickness, and the discomfort surface area. Four general categories of helmets were tested. The cycling helmet (SH2) had a hard shell and firm foam (and soft comfort pads). MH1 and MH4 had hard shells and soft foams. MH6 had a flexible shell and a soft, energy-absorbing material. The remaining helmets just had soft foam layers.

**TABLE 1:** Results for non-destructive characterisation of helmets.

Test code	Mass (grams)	Descriptor		Foam properties				Discomfort area after fitting (cm^2^)
		Shell material	Foam	Shore O hardness		Thickness (mm)		
				Outer/inner	Mean	Front	Side	
MH1	353	Hard	Soft	-	29	33	22	3.05
MH2	129	-	Soft	36/37	36.5	35	27	0.52
MH3	110	-	Soft	38/33	35.5	16	16	0.00
MH4	231	Hard	Soft	-	20	12	12	0.95
MH5	276	-	Soft	17/14	15	15	15	0.95
MH6	272	Flexible*	Soft	-	27	8	7	0.00
SH1	81	-	Soft	19/15	17	13	13	0.03
SH2	197	Hard	Firm	-	-	23	19	0.09

* measured as Shore 56D.

### Kinematic response

#### Peak linear acceleration

**FIGURE 2:**
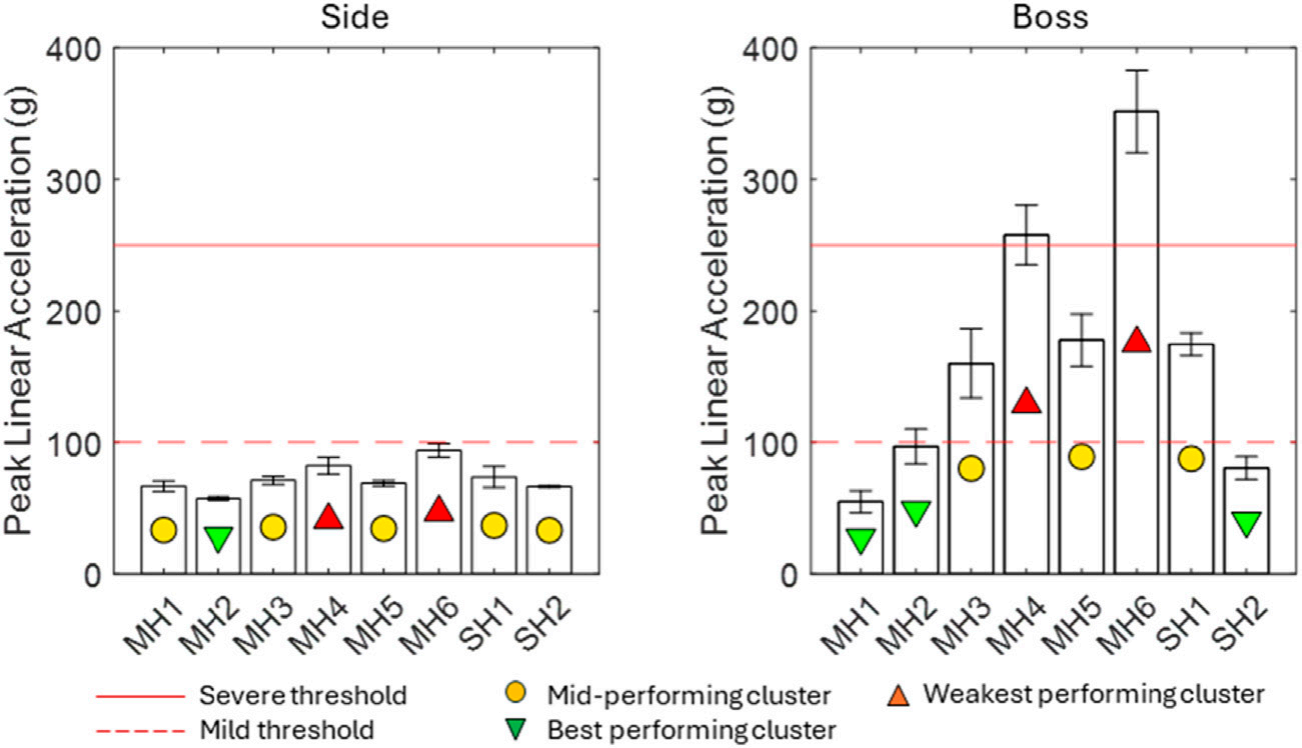
PLA results for eight helmets, including a typical pass/fail threshold from test standards that represents severe injury (red dash) and a typical threshold for moderate injury from the literature (orange dash).

Figure 2 presents the magnitude response of PLA. For context, the typical pass/fail criterion of 250 g in regulatory standards (e.g., cycling) has been included, which correlates with a 40% risk of skull fractures (Mertz et al., 1997). A threshold of 100 g, which is typical of mTBI thresholds in the literature, is also plotted (Viano, 2005; McIntosh et al., 2014). The PLA ranged from 55 g to 352 g. In seven out of eight cases, the PLA magnitude at the boss location was greater than that in the frontal location. The magnitude at the boss location was between 40% and 350% greater for most models. SH2 and MH1 were least affected by impact location. MH2 was the only model in the best-performing cluster for the side impact and boss impact locations, making it the best-performing model according to the PLA in the two tested locations. Every side impact was below the 100 g threshold, but only MH1 and SH2 were below 100 g for the boss impact. Both MH4 and MH6 exceeded the 250 g threshold at the boss location.

#### Peak angular velocity

**FIGURE 3:**
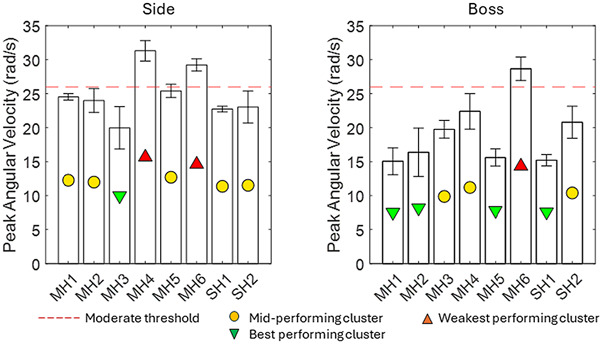
PAV results for eight helmets, including a typical threshold for moderate injury from the literature (orange).

Figure 3 presents the results for PAV at both impact locations. For context, the plots include a threshold of 26 rad/s, representing an approximate 50% risk of mTBI, as surmised from several sources (Rowson et al., 2012; Campolettano et al., 2020). The PAV ranged from 15.0 to 31.3 rad/s across all impacts. In six out of eight cases, the PAV magnitude was lower for impacts at the boss location than that at the side location; MH3 and MH6 were similar in both locations. MH4 and MH6 exceeded the mTBI threshold for the side impact, and MH6 exceeded the threshold for the boss impact.

#### Peak angular acceleration

**FIGURE 4:**
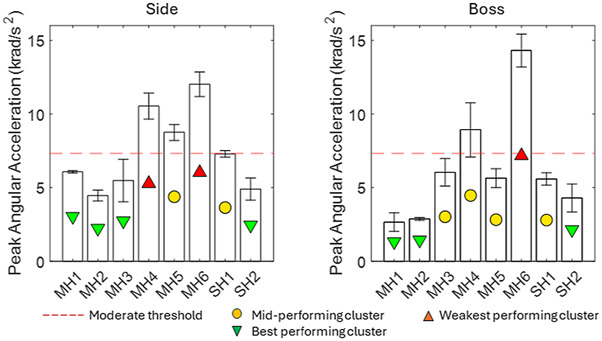
PAA results for eight helmets, including a typical threshold for moderate injury from the literature (orange).

Figure 4 presents the results for PAA across both impact locations. For context, a threshold of 7.5 krad/s^2^ for mTBI has been surmised from several sources (Broglio et al., 2010; McIntosh et al., 2014). The PAA ranged from 2.6 rad/s^2^ to 14.3 rad/s^2^. SH2, MH3, and MH4 performed similarly across the two impact locations. MH1, MH2, and SH2 were the best-performing helmets at both impact locations. At the frontal location, only MH4 and MH6 exceeded the proposed threshold value. At the side location, MH4, MH5, and MH6 exceeded the proposed threshold value.

#### Head injury criterion

**FIGURE 5:**
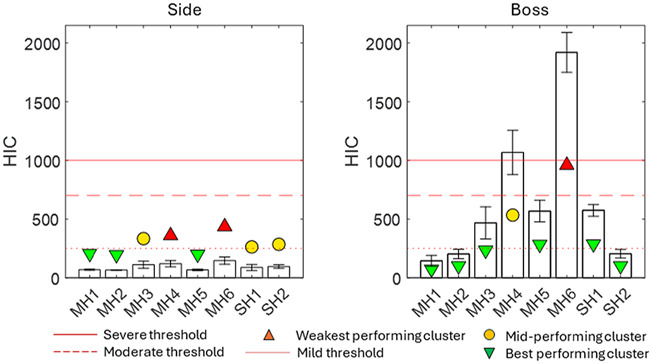
HIC results for eight helmets, including thresholds for severe (red), moderate (orange), and mild (blue) injuries from the literature.

Figure 5 presents the results for HIC, including three injury thresholds for context. A magnitude of 1000 corresponds to an 18% chance of AIS4+ injury and a 90% chance of AIS2+ injury (Mackay, 2007); a magnitude of 700 corresponds to a 5% chance of AIS4+ injury and a 32% chance of AIS2+ injury; and a magnitude of 250 corresponds to a 5% chance of AIS2+ injury and typically correlates with concussion (Viano, 2005). HIC results ranged from 65 to 145 for side impacts and from 144 to 1920 for boss impacts. The boss impact was the most severe impact location in every instance, with a magnitude between 111% and 1,223% greater than the side impact (Figure 5).

MH6 was in the weakest-performing cluster for both locations, making it the overall weakest-performing helmet by HIC. MH1 and MH2 were the best all-round performing helmets.

#### Brain injury criterion

**FIGURE 6:**
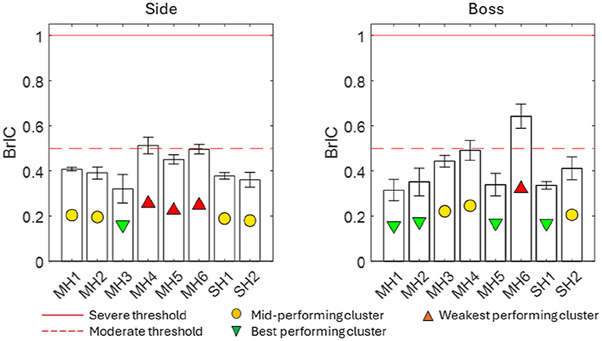
Brain injury criterion results for the eight helmets, including thresholds for severe and moderate injury from the literature.

Figure 6 presents the results for BrIC, including a threshold of 1.0 for serious injury (50% probability of AIS4+) and a threshold of 0.5 for moderately severe injury (50% probability of AIS2+). BrIC ranged from 0.32 to 0.64. In the boss impact, MH6 was the weakest-performing helmet and was amongst the weakest-performing helmets at the side location. None of the impacts exceeded the threshold of 1.0. MH6 and MH4 met or exceeded the threshold of 0.5 at both locations.

#### DAMAGE as a predictor of MPS

**FIGURE 7:**
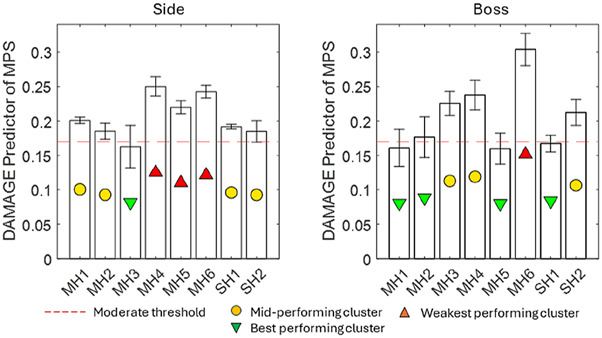
DAMAGE injury criterion results as a predictor of MPS for eight helmets, including a typical threshold for moderate injury from the literature (orange).

Figure 7 presents the results for DAMAGE as a predictor of MPS. An injury threshold of 0.17 MPS was used as a benchmark for moderate injury severity, as surmised from several sources in the literature (Willinger and Baumgartner, 2003; Zhang et al., 2004; Kleiven, 2007; Fahlstedt et al., 2022). MPS ranged from 0.16 to 0.30. MH6 was the all-round lowest-performing helmet. Overall, the range from the best-performing helmets to the mid-performing helmets in the side impact was small. The best-performing helmets for the boss impact were MH1, MH2, MH5, and SH1. All helmets exceeded the threshold of 0.17 MPS in at least one impact location.

#### Contact pressure

**FIGURE 8:**
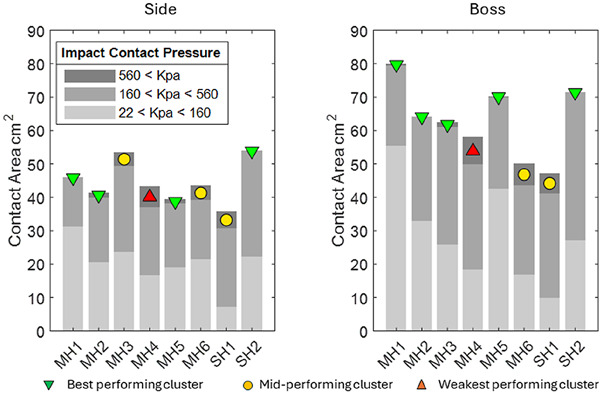
Results for contact pressure area measured within specified ranges during impact.

Figure 8 presents the contact pressure measurements obtained on the surface of the skin at the targeted impact locations. Each bar aggregates the surface area measured within three magnitude ranges representing low, moderate, and high pressures. The cluster ranking is shown with coloured markers and is based only on the magnitude of the most severe pressure range (>560 kPa). Helmets MH1 and SH2 were the all-round best-performing helmets with the smallest high-pressure region. SH2 had a large medium-pressure region. This is in contrast with the mid- and weakest-performing helmets, which exhibit between 4.2 and 8.3 cm^2^ of high contact pressure. MH4 was found to be the weakest-performing helmet.

#### Combined results

**FIGURE 9:**
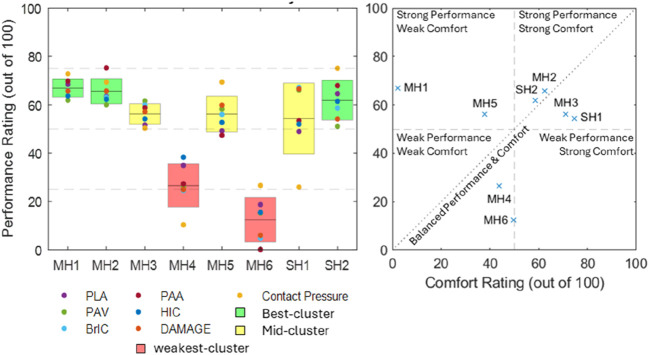
Results for net performance rating per helmet (left) and net performance rating plotted against net comfort rating (right).

Figure 9 (left) presents the net performance rating calculated per helmet for the performance metrics presented above. For each helmet, the performance rating for each metric is plotted individually (round markers). Each helmet has a bar that represents the combined net performance and uncertainty between the metrics (±1 standard deviation). These are clustered by mean into best-, mid-, and weakest-performing helmet categories. Figure 9 (right) shows plots of the net performance rating versus the net comfort rating. From the net performance ratings (Figure 9, left), the best-performing helmets were MH1, MH2, and SH2. MH1 had the smallest range, indicating that it was the most consistent top performer across the metrics presented. MH4 and MH6 were the weakest-performing helmets (26.6 and 12.4, respectively), both significantly below the averages for the mid- and best-performing clusters. For the plot of net performance rating against comfort rating (Figure 8, right), the best-performing helmets were SH2, MH2, MH3, and SH1 (top right quadrant). MH3 and SH1 scored better in terms of comfort than performance, and MH2 and SH2 performed better in terms of performance than comfort. MH1 performed well but had the lowest rating for comfort by a significant margin.

## Discussion

### Helmet characterisation

None of the soft helmets exhibited dramatically different Shore hardness values internally and externally, and the variation observed was attributed to measurement uncertainty due to challenging curved geometry. Most helmets had the same liner thickness at the front and side, but those that differed had an increased thickness at the front (MH1, MH2, and SH2). This was perhaps due to the perceived increased frequency of forward falls or aesthetic ‘bulkiness’ when viewed front-on. The SHs were generally lighter than the MHs (mean 139 g compared to 228 g), although individual examples, such as MH2 and MH3, break this trend. This lightweight nature of SHs is driven by performance demands but inherently results in a better comfort score when considering product weight as a proxy measure.

The discomfort area was correlated with the construction type. The two helmets with close-fitting foam layers and hard shells had the largest discomfort areas (MH1 and MH4). The soft construction helmets ranged from 0 to 0.96 cm^2^, and the discomfort area was anecdotally correlated to material stiffness when considering the force required for stretching the helmet over the headform. MH2 had the same internal dimensions as MH1, but the absence of a hard shell allowed easier stretching over the headform and resulted in a much smaller measured discomfort region. MH5 had a ‘stretchy’ construction and, therefore, imparted a larger but low magnitude (more distributed) discomfort area to the head. Whether this level of pressure would be acceptable to a patient is unknown. Despite being the recommended size for the headform, MH6 and MH3 were visually oversized and, therefore, did not impart any discomfort region. Similarly, SH1 (bicycle helmet) uses a lightweight adjustable retention system that anchors just below the equator of the head, meaning that most of the applied pressure holding SH1 in place was below the instrumented region.

### Impact kinematics

The performance of MHs varied significantly by model, highlighting that patients would receive different levels of protection depending on which MH was prescribed. The difference between the best- and worst-performing helmets ranged from a 90% increase to a 2,844% increase across each metric and both impact locations. The largest performance gaps between the best- and worst-performing helmets were observed for HIC (2844%), PLA (542%), and PAA (441%). SHs were consistently amongst the best-performing helmets in this study. MH1, MH2, and MH3 were consistently among the best-performing MHs and were comparable to the SHs. In contrast, MH4 and MH6 were consistently amongst the worst-performing helmets, and in 7 out of 16 metric/location combinations, they produced kinematic magnitudes at least twice as high as the best-performing helmets.

For all helmets, the PLA magnitude was found higher at the boss location, with all side impacts resulting in a PLA below the 100 g threshold. In contrast, the PAV magnitude was smaller at the boss location than that at the side for 5/8 helmets. This combination indicates that the vector of the side impact must have been further offset from the centre of mass of the headform to generate less linear and more angular kinematics. Linear and angular kinematics are generally associated with different injury pathologies (Kleiven, 2013); therefore, the inclusion of impacts that generate both allows the results of this study to be more representative of likely real-world impact scenarios. At the boss location, three helmets (MH3, MH5, and SH1) exceeded the 100 g threshold and two (MH4 and MH6) exceeded the 250 g threshold for severe injury. This indicates that these two helmets would not pass a regulatory standard drop test from sports (Emsley et al., 2024) despite the significantly lower drop height in this study compared to sports. The best-performing helmets (MH1, SH2, and MH2 borderline) satisfied the more conservative threshold of 100 g at both locations.

MH3, SH1, and SH2 were most significantly below the literature threshold for moderate injury when averaged across both locations, while MH1, MH2, and MH5 were significantly below this threshold at the boss location. The PAA magnitude was smaller at the boss location for 5/8 helmets, demonstrating that although it followed the same general trend as the PAV, two helmets exhibited a significant change. For PAA, only MH1, MH2, MH3, and SH2 satisfied the literature threshold for moderate injury at both locations. SH1 satisfied this only for the boss location. In contrast to PAV, BrIC showed no systematic differences between impact locations. This was because the boss impact generated more axial rotation than the side impact, and BrIC suggests that human brain injury is more sensitive to axial rotation (Takhounts et al., 2013). HIC exhibited the largest range between the best- and worst-performing helmets at the boss location. The linear acceleration time history shows not only a greater magnitude of PLA but also, on average, between 7% and 14% longer contact times. For the side impact, none of the impacts exceeded the 250 HIC threshold and, therefore, predicted a low risk of AIS2+ injury. At the boss location, MH4 and MH6 exceeded the 1000 threshold, indicating a high risk of AIS4+ injury. MH3, MH5, and SH1 exceeded the 250 HIC threshold, which represents a moderate risk of moderate injury (AIS2+).

There are two existing studies of MH performance in the literature (Martel et al., 2021; Barrett et al., 2022). Neither reflected the range of products available to the NHS nor gave specific consideration for patients post-DC surgery (including the use of less relevant impact locations). Martel et al. (2021) considered only PLA and used the guided drop test method. The range of results for the front boss impacts in this study (55–352 g) agreed well with that for their frontal location (145–340 g). Barrett et al. (2022) reported PLA in the range of 200–500 g, which exceeded the range of results from this study and is explained by their more severe reverse pendulum impact methodology. The magnitude differences from best to worst (∼300 g) were similar to those observed in this study. Barrett et al. (2022) reported PAA in the range of 11–21 krad/s^2^ compared to the results of this study, which ranged from 3 to 14 krad/s^2^. Similar to PLA, the magnitude difference can be attributed to the difference in methodology; however, PAA also exhibited a similar magnitude range, from best to worst (∼10 krad/s^2^). Barrett et al. (2022) reported HIC magnitudes ranging from 100 to 550, which contrasts with the results of this study, where they ranged from 65 and 1920. In this study, only two results exceeded the maximum reported by Barrett et al. (2022) (MH3 and MH6), showing reasonable agreement between studies. It is possible that the soft tissue of the LU1.1 headform elongated the contact duration, leading to increased HIC for similar PLA. There was no overlap between the products tested by Martel et al. (2021), Barrett et al. (2022), and this study, and due to the specific consideration for DC patients, this study’s side impact was much more relevant than their rear impact because the side is the most common DC location. The results for helmets MH1, MH2, and SH2 in this study fell below the PLA range reported by Martel et al. (2021), indicating the value of testing helmets specific to a healthcare provider or region.

### Contact pressure

Contact pressure was measured locally at the impact locations and was primarily used to estimate the area that exceeded a threshold of 560 kPa to indicate possible bruising (Jones et al., 2007). MH1, MH2, MH5, and SH2 were the best-performing helmets across both locations, with SH2 being the only helmet that never exceeded 560 kPa. MH2 and MH5 did not exhibit any area exceeding 560 kPa for the side impact but consistently exhibited a small region exceeding this threshold for the boss location. The contact area for the lower pressure ranges (22–160 and 160–560 kPa) highlighted the mechanism of intervention that made MH1, MH2, MH5, and SH2 the most effective helmets according to the contact pressure. At the side location, these were amongst the smallest areas exceeding 560 kPa, but the largest areas exceeding 22 kPa showed that they were effective in spreading the load over the instrumented region (and also perhaps away from the instrumented region). At the boss location, this was apparent for MH1 and SH2, indicating that the hard-shelled helmets had a better ability to distribute the load for the boss impact. The reduced overall contact areas at the boss location showed that this impact was inherently more focal due to head geometry and soft tissue thickness, which seemingly reduced the ability of MH2 and MH5 to spread the load. Nonetheless, the magnitude of the area in which MH2 and MH5 exceeded 560 kPa was smaller than that of the other MHs. MH4, MH6, and SH1 were consistently the worst-performing helmets, which is likely due to their thinner and softer functional foam layers.

### Overall performance

Figure 9 shows the combination of all metrics into a net performance rating and a net comfort rating relative to the range of results within this study and presents the rank order of the tested helmets. The top right quadrant represents the best overall helmets and includes MH2, MH3, SH1, and SH2. Helmets closest to the diagonal line had the best balance between comfort and performance, which were MH2, SH2, MH3, and SH2, in order of descending proximity. Helmets outside this quadrant highlight at least one significant deficiency. MH1 and MH5 were in the upper left quadrant, representing above-average impact performance but potentially flawed comfort, which might discourage DC patients from wearing them. This was more significant for MH1 than MH5. The weak performance ratings of MH4 and MH6 were not redeemed by the comfort rating, as they were also below average.

Overall, MH2 scored the highest in the combined comfort and performance rating, while MH3 exhibited the best comfort with slightly lower performance, making it the second choice. Neither MH2 nor MH3 was constructed with a hard shell, making them lightweight while also preventing high localised pressure on the craniectomy site during general wear. This was exemplified by the good comfort scores for these helmets. The closeness of fit did not seem to influence the ratings as MH2 was amongst the tightest fitting helmets, and in contrast, MH3 was amongst the looser fitting helmets. With respect to their performance, both utilised foam that was amongst the stiffest tested (35–38 Shore O). This enabled them to perform well in the drop test without an additional hard outer shell. MH2 used a thicker foam (35 mm compared to 16 mm at the front) and outperformed MH3 in the front-boss impact (all metrics), where the impact site was more focal. The side impact was more diffuse, and the performance was relatively equal for MH2 (better for PLA, PAA, and HIC) and MH3 (better for PAV, BrIC, and DAMAGE). MH1 exhibited marginally better impact performance than MH2 but at a significant comfort disadvantage. It was found to be almost three times heavier than both MH2 and MH3 and exerted the largest pressure area on the craniectomy site at rest, likely due to the rigid, hard outer shell construction.

### Limitations

Contact pressure measurement exceeding the threshold of 560 kPa was a valuable finding because it can be correlated with possible bruising. Unfortunately, delineation above 560 kPa was not possible with the available equipment, but 560 kPa was an appropriate threshold of concern for delicate brain tissue. The pressure measurement can be improved with faster sampling and a larger pressure range. Another limitation in the pressure measurement was the lack of anatomical detail related to DC surgery; if the surrogate headform had an instrumented biofidelic DC, the findings would be more valid. A limitation of this study was that the MHs in this study were limited to those available to the NHS rehabilitation unit in Cambridge.

## Summary

The first recommendation is that MH2 and MH3 are the best MHs available. In terms of predicted comfort and impact performance, they were comparable with the SH examples tested, in which standards testing has been widely shown to be adequate for demanding sports scenarios. A second recommendation is to select among MH2, MH3, or MH1 based on the individual patient’s needs, with MH1 being a ‘high-risk’ option with maximal impact performance. MH design should learn from SH design, and clinicians should explore the regulatory possibility of using an SH in an MH context, especially if patients perceive the SHs to be more aesthetically agreeable or the SHs are significantly cheaper.

This study highlighted the wide variability in the performance of MHs and informed clinical decisions made by healthcare professionals. The consideration of DC surgery as a specific use case contributed significantly to new findings not otherwise addressed in the literature. Kinematics showed great agreement in rating MH6 as the weakest-performing helmet by a significant margin. However, contact pressure measurements showed that MH4 and SH1 were equally weak. The fact that the pressure measurement showed the most atypical trends compared to the other metrics highlights the value of its inclusion. This study’s exploration of DC-specific metrics was limited to available cost-effective measurements. More work is required to focus on the factors more specific to the risk of falling after DC surgery. Aesthetics and comfort are major barriers for DC patients wearing MHs and participating in rehabilitation (Mee et al., 2022b). Therefore, more consideration is required for rating the aesthetic quality of MHs and a human perception study into the comfort of MHs would be more appropriate than the simplistic perspective on comfort presented in this study. If a patient chooses not to wear the MH, it cannot perform its function, and if wearing the MH causes a patient to become more recluse due to social inhibition from aesthetics, this will hinder recovery. The fact that two of the MHs were consistently and very significantly deficient compared to SHs and the other MHs highlights the urgent need for standardisation and certification in this sector for patient safety.

## Data Availability

The raw data supporting the conclusions of this article will be made available by the authors, without undue reservation.

## Abbreviations

DC, decompressive craniectomy; MH, medical helmet; SH, sports helmet; HIC, head injury criterion; BrIC, brain injury criterion; PLA, peak linear acceleration; PAV, peak angular velocity; PAA, peak angular acceleration.

## References

[B1] Barrett B. Peterson M. J. Phillips S. L. Lloyd J. Cowan L. Friedman Y. (2022). Evaluation of protective properties of commercially available medical helmets: are medical helmets protective? J. Patient Saf. 18, e205–e210. 10.1097/PTS.0000000000000736 34951609

[B2] Broglio S. P. Schnebel B. Sosnoff J. J. Shin S. Feng X. He X. (2010). Biomechanical properties of concussions in high School football. Med. Sci. Sports Exerc 42, 2064–2071. 10.1249/MSS.0b013e3181dd9156 20351593 PMC2943536

[B3] Campolettano E. T. Gellner R. A. Smith E. P. Bellamkonda S. Tierney C. T. Crisco J. J. (2020). Development of a concussion risk function for a youth population using head linear and rotational acceleration. Ann. Biomed. Eng. 48, 92–103. 10.1007/s10439-019-02382-2 31659605 PMC6928097

[B4] David A. Vassilvitskii S. (2007). “K-means++: the advantages of careful seeding,” in Soda ‘07: proceedings of the eighteenth annual ACM-SIAM symposium on discrete algorithms, 1027–1035.

[B5] Department of Health and Social Care (PHOF) (2019). Falls: applying all our health.

[B6] Emsley B. Farmer J. Sherratt P. Goodall P. Jackson T. West A. (2024). An overview of the test methodology used in current cycling helmet standards and literature. Int. J. Impact Eng. 188, 104928. 10.1016/j.ijimpeng.2024.104928

[B7] England R. (2025). Advanced biofidelic headforms for high fidelity re-enactment of real-world head impacts in cricket.

[B8] Evans D. W. Mear E. Neal B. S. Waterworth S. Liew B. X. W. (2024). Words matter: effects of instructional cues on pressure pain threshold values in healthy people. Musculoskelet. Sci. Pract. 73, 103150. 10.1016/j.msksp.2024.103150 39089120

[B9] Fahlstedt M. Meng S. Kleiven S. (2022). Influence of strain post-processing on brain injury prediction. J. Biomech. 132, 110940. 10.1016/j.jbiomech.2021.110940 35065410

[B10] Gabler L. F. Crandall J. R. Panzer M. B. (2019). Development of a second-order system for rapid estimation of maximum brain strain. Ann. Biomed. Eng. 47, 1971–1981. 10.1007/s10439-018-02179-9 30515603

[B11] Jadischke R. Viano D. C. Dau N. King A. I. McCarthy J. (2013). On the accuracy of the Head Impact Telemetry (HIT) System used in football helmets. J. Biomech. 46, 2310–2315. 10.1016/j.jbiomech.2013.05.030 23891566

[B12] Jones D. Kilgour R. Comtois A. (2007). Test-retest reliability of pressure pain threshold measurements of the upper limb and torso in young healthy women. J. Pain 8, 650–656. 10.1016/j.jpain.2007.04.003 17553750

[B13] Kleiven S. (2007). Predictors for traumatic brain injuries evaluated through accident reconstructions. Stapp Car Crash J. 51, 81–114. 10.4271/2007-22-0003 18278592

[B14] Kleiven S. (2013). Why most traumatic brain injuries are not caused by linear acceleration but skull fractures are. Front. Bioeng. Biotechnol. 1, 15–5. 10.3389/fbioe.2013.00015 25022321 PMC4090913

[B15] Mackay M. (2007). The increasing importance of the biomechanics of impact trauma. Sadhana 32, 397–408. 10.1007/s12046-007-0031-9

[B16] Maquet D. Croisier J. Demoulin C. Crielaard J. (2004). Pressure pain thresholds of tender point sites in patients with fibromyalgia and in healthy controls. Eur. J. Pain 8, 111–117. 10.1016/S1090-3801(03)00082-X 14987620

[B17] Martel D. R. Tanel M. R. Laing A. C. (2021). Impact attenuation provided by older adult protective headwear products during simulated fall-related head impacts. J. Rehabil. Assist. Technol. Eng. 8, 20556683211050357. 10.1177/20556683211050357 34877017 PMC8645304

[B18] Martin P. G. Hall G. W. Crandall J. R. Pilkey W. D. (1998). Measuring the acceleration of a rigid body. Shock Vib. 5, 211–224. 10.1155/1998/134562

[B19] McIntosh A. S. Patton D. A. Fréchède B. Pierré P.-A. Ferry E. Barthels T. (2014). The biomechanics of concussion in unhelmeted football players in Australia: a case–control study. BMJ Open 4, e005078. 10.1136/bmjopen-2014-005078 PMC4039841 24844272

[B20] Mee H. Anwar F. Timofeev I. Owens N. Grieve K. Whiting G. (2022a). Cranioplasty: a multidisciplinary approach. Front. Surg. 9, 864385. 10.3389/fsurg.2022.864385 35656088 PMC9152220

[B21] Mee H. Clement C. Anwar F. Whiting G. Timofeev I. Helmy A. (2022b). Exploring the experiences and challenges for patients undergoing cranioplasty: a mixed-methods study protocol. BMJ Open 12, e048072. 10.1136/bmjopen-2020-048072 PMC9036468 35459659

[B22] Mertz H. Prasad P. Irwin A. (1997). “Injury risk curves for children and adults in frontal and rear collisions,” in Proceedings of the 41th stapp car crash conference (Lake Buena Vista). 10.4271/973318

[B23] Moon J. W. Hyun D. K. (2017). Decompressive craniectomy in traumatic brain injury: a review article. Korean J. Neurotrauma 13, 1. 10.13004/kjnt.2017.13.1.1 28512611 PMC5432443

[B24] Morse J. M. Gervais P. Pooler C. Merryweather A. Doig A. K. Bloswick D. (2015). The safety of hospital beds: ingress, egress, and in-bed mobility. Glob. Qual. Nurs. Res. 2, 2333393615575321. 10.1177/2333393615575321 28462302 PMC5371163

[B25] Newman J. A. (1980). Head injury criteria in automotive crash testing. 10.4271/801317

[B26] Rowson S. Duma S. M. Beckwith J. G. Chu J. J. Greenwald R. M. Crisco J. J. (2012). Rotational head kinematics in football impacts: an injury risk function for concussion. Ann. Biomed. Eng. 40, 1–13. 10.1007/s10439-011-0392-4 22012081 PMC10465647

[B27] Silva A. C. V. De Oliveira Farias M. A. Bem L. S. Valença M. M. De Azevedo Filho H. R. C. (2020). Decompressive craniectomy in traumatic brain injury: an institutional experience of 131 cases in two years. Neurotrauma Rep. 1, 93–99. 10.1089/neur.2020.0007 34223535 PMC8240881

[B28] Takhounts E. G. Craig M. J. Moorhouse K. Mcfadden J. Hasija V. (2013). Development of brain injury criteria (BrIC). Stapp Car Crash J. 57, 243–266. 10.4271/2013-22-0010 24435734

[B29] Trueba J. H. Gasparini F. (2021). Pressure pain threshold values obtained through algometers. Rev. Mex. De. Ing. Biomed. 10.17488/RMIB.42.2.11

[B30] Viano D. C. (2005). “Head impact biomechanics in sport,” in IUTAM symposium on impact biomechanics: from fundamental insights to applications (Berlin/Heidelberg: Springer-Verlag), 121–130. 10.1007/1-4020-3796-1_12

[B31] Willinger R. Baumgartner D. (2003). Human head tolerance limits to specific injury mechanisms. Int. J. Crashworthiness 8, 605–617. 10.1533/ijcr.2003.0264

[B32] Zhang L. Yang K. H. King A. I. (2004). A proposed injury threshold for mild traumatic brain injury. J. Biomed. Eng. 126, 226–236. 10.1115/1.1691446 15179853

